# Diarrheal-associated gut dysbiosis in cancer and inflammatory bowel disease patients is exacerbated by *Clostridioides difficile* infection

**DOI:** 10.3389/fcimb.2023.1190910

**Published:** 2023-07-27

**Authors:** Maria Kulecka, Natalia Zeber-Lubecka, Aneta Bałabas, Paweł Czarnowski, Katarzyna Bagińska, Maria Głowienka, Anna Kluska, Magdalena Piątkowska, Michalina Dąbrowska, Edyta Waker, Michał Mikula, Jerzy Ostrowski

**Affiliations:** ^1^ Department of Gastroenterology, Hepatology and Clinical Oncology, Centre of Postgraduate Medical Education, Warsaw, Poland; ^2^ Department of Genetics, Maria Sklodowska-Curie National Research Institute of Oncology, Warsaw, Poland; ^3^ Department of Biochemistry, Radioimmunology and Experimental Medicine, The Children’s Memorial Health Institute, Warsaw, Poland; ^4^ Department of Clinical Microbiology, Maria Sklodowska-Curie National Research Institute of Oncology, Warsaw, Poland

**Keywords:** diarrhea, gut dysbiosis, inflammatory bowel disease, *Clostridioides difficile*, microbiome, metabolites, CDI

## Abstract

**Introduction:**

Low diversity gut dysbiosis can take different forms depending on the disease context. In this study, we used shotgun metagenomic sequencing and gas chromatography–mass spectrometry (GC-MS) to compared the metagenomic and metabolomic profiles of *Clostridioides (Clostridium) difficile* diarrheal cancer and inflammatory bowel disease (IBD) patients and defined the additive effect of *C. difficile* infection (CDI) on intestinal dysbiosis.

**Results:**

The study cohort consisted of 138 case-mix cancer patients, 43 IBD patients, and 45 healthy control individuals. Thirty-three patients were also infected with *C. difficile*. In the control group, three well-known enterotypes were identified, while the other groups presented with an additional *Escherichia*-driven enterotype. Bacterial diversity was significantly lower in all groups than in healthy controls, while the highest level of bacterial species richness was observed in cancer patients. Fifty-six bacterial species had abundance levels that differentiated diarrheal patient groups from the control group. Of these species, 52 and 4 (*Bacteroides fragilis*, *Escherichia coli*, *Klebsiella pneumoniae*, and *Ruminococcus gnavus*) were under-represented and over-represented, respectively, in all diarrheal patient groups. The relative abundances of propionate and butyrate were significantly lower in fecal samples from IBD and CDI patients than in control samples. Isobutyrate, propanate, and butyrate concentrations were lower in cancer, IBD, and CDI samples, respectively. Glycine and valine amino acids were over- represented in diarrheal patients.

**Conclusion:**

Our data indicate that different external and internal factors drive comparable profiles of low diversity dysbiosis. While diarrheal-related low diversity dysbiosis may be a consequence of systemic cancer therapy, a similar phenotype is observed in cases of moderate to severe IBD, and in both cases, dysbiosis is exacerbated by incidence of CDI.

## Introduction

In cancer patients, diarrhea can have both a noninfectious and infectious etiology. Noninfectious etiologies include cytotoxic chemotherapy or radiation-related mucosal injury, leading to altered gastrointestinal motility, which can be associated with secretory or osmotic diarrhea (Schultsz et al., 2000; Toprak et al., 2006; Lu et al., 2017; Hebbelstrup Jensen et al., 2018; Mobeen et al., 2018; The et al., 2018; Zhang et al., 2018; Li et al., 2021). Furthermore, antibiotics commonly used as part of routine cancer therapeutic strategies, such as clindamycin, cephalosporins, and fluoroquinolones, alter composition of the enteric microbiome and are a significant risk factor for *Clostridioides (Clostridium) difficile* infection (CDI) (Battaglioli et al., 2018). Most cases of infectious diarrhea in cancer patients are related to CDI, and these infections are the main cause of healthcare-associated diarrhea (Wick and Sears, 2010; Kriss et al., 2018; Zhai et al., 2022). CDI risk is eight to ten times higher during antimicrobial treatment regimens and the 4 weeks after discontinuing therapy, and the risk remains three times higher for a further 2 months (Han et al., 2022). Due to frequent or prolonged hospitalization and extensive antibiotic treatment, the rate of CDI in cancer patients is twice as high as that in other hospital patients (Denève et al., 2009). Together, diarrhea in cancer patients has multiple etiologies, depending on both therapeutic side effects and cancer therapy outcomes (Lu et al., 2017) that are accompanied by changes in the gut microbiota composition and/or a shift in its local distribution, a phenomenon termed dysbiosis. Although cases of dysbiosis are highly variable depending on disease context (Chung The and Le, 2022), low diversity of the microbiota and increased proportions of facultative anaerobes, such as *Proteobacteria* and *Bacilli*, are typically exacerbated by CDI, CDI-negative antibiotic-associated and nosocomial diarrhea, inflammatory bowel disease (IBD), and other inflammation-related diarrhea (Karen and John, 2011; Antharam et al., 2013; Theriot and Young, 2015; Mekonnen et al., 2020).

IBD, Crohn’s disease (CD), and ulcerative colitis (UC) are classic polygenic disorders, in which multiple genetic factors and their interaction with the intestinal microbiota drive several intestinal immunopathologies (Alonso et al., 2012; Alasmari et al., 2014). As with other systemic inflammatory conditions, the intestinal microbiota is dysbiotic in IBD patients (Alonso et al., 2012; Fuereder et al., 2016; Hebbard et al., 2016; Park et al., 2016; Cornely et al., 2018; Czepiel et al., 2019; Shoaei et al., 2019). Although the drivers of IBD-related dysbiosis remain unclear, patients with diarrhea routinely undergo microbiological testing to identify the presence of *C. difficile* toxins (Deleu et al., 2021). Patients with IBD and associated *C. difficile* infection have more pronounced dysbiosis with higher frequencies of *Ruminococcus gnavus* and *Enterococci*, and lower levels of *Blautia* and *Dorea*. These findings suggest a close relationship between perturbations of the intestinal microbiome and inflammation-related diarrhea in patients with IBD, particularly those with CDI (Alasmari et al., 2014).

The intestinal microbiota can both inhibit and promote diarrhea by generating endogenous metabolites and microbial products, such as short-chain fatty acids (SCFAs, including butyrate, acetate, and propionate), amino acids, secondary bile acids, and lipopolysaccharides. Low diversity dysbiosis is characterized by a decreased abundance of SCFA-producing bacteria (Hugerth et al., 2020; Rouhani et al., 2020) associated with lower concentrations of SCFA in fecal samples. The antagonistic effects of SCFAs on intestinal dysbiosis are mediated by interactions with intestinal epithelial cells (IECs) and immune cells (Carroll and Bartlett, 2011). Butyrate is utilized as an energy source for colonocytes, enhances the integrity of IECs, increases mucin production, and induces differentiation of naive T cells (Salvi and Cowles, 2021). Acetate and propionate promote anti-inflammatory processes by regulating functions of leukocytes and endothelial cells, which include production of cytokines (TNF-α, IL-2, IL-6 and IL-10), eicosanoids and chemokines (e.g., MCP-1 and CINC-2). However, pro-inflammatory action of SCFAs has been also recognized in some conditions (Tedelind et al., 2007; Vinolo et al., 2011). To end this, the levels of SCFAs correlate with variations in the gut microbiota (Ross et al., 2016; Battaglioli et al., 2018; Zhang et al., 2019) and can modulate the severity of *C. difficile* infection or directly inhibit growth of the bacteria (Ramirez et al., 2020; Chandra et al., 2021).

In this study, we used shotgun metagenomic sequencing and GC/MS metabolomics to compare the microbiota profiles and metabolite levels of hospitalized cancer patients with healthcare-associated diarrhea, patients with IBD, and healthy controls. Our data define a role of CDI in modulating low diversity dysbiosis.

## Materials and methods

### Patients

Between January and June 2021, a total of 226 participants were enrolled in this study, including 138 case-mix cancer patients, 43 patients with moderate to severe IBD, and 45 healthy control individuals.

The cancer patient group consisted of 53 males and 85 females (median age, 64 years; range, 15–89 years), hospitalized for a wide range of neoplastic diseases and undergoing different treatment regimens (**Table 1**). All cancer patients exhibited healthcare-associated diarrhea, which was defined as the passage of three or more loose or liquid stools per day.

**Table 1:** Clinical presentation of patients enrolled in this study and their treatment regimens.

Type of neoplastic disease (number of patients)	Current treatment or treatment in the last 2 months (number of treated patients)			
	Antibiotics	ChT	RT	IT
**Colorectal cancer (n = 13)**	4	7	3	0
**Gastric cancer (n = 7)**	2	5	1	1
**Other cancers of the digestive system (n = 9)**	2	7	4	0
**Case-mix lymphomas (n = 45)**	10	42	6	5
**Prostate cancer (n = 6)**	1	3	4	0
**Urinary tract neoplasms (n = 6)**	4	3	0	0
**Lung cancers (n = 9)**	0	7	5	3
**Gynecological tumors (n = 19)**	4	13	4	1
**Breast cancers (n = 5)**	2	4	0	0
**Melanomas (n = 19)**	6	3	1	8
**Total (n = 138)**	35	94	28	18

ChT, chemotherapy; RT, radiotherapy; IT, immunotherapy.

The IBD patient group consisted of 26 males and 17 females (median age, 35 years; range, 14–79) with a diagnosis of UC (n = 34) or CD (n = 9) according to clinical, radiological, endoscopic, and histological criteria in line with the European Crohn’s and Colitis Organization (ECCO) guidelines. In all cases, the Crohn’s disease activity index (CDAI) or the ulcerative colitis activity index (UCAI) established moderate or severe IBD. Patients with IBD were recruited during a course of hospital treatment or during a scheduled visit to the outpatient department at the Department of Gastroenterology. One patient with IBD received antibiotic treatment in the 2 months prior to being enrolled in the study. A summary of the current treatment regimens is provided in **Table 2**.

**Table 2:** Summary of current treatment regimens used for patients with inflammatory bowel disease.

IBD patients (number of patients)	Current treatment (number of patients)			
	5-ASAs	Glucocorticoids	Immunosuppressants	Biologic therapy
**Ulcerative colitis (n = 9)**	9	4	4	5
**Crohn’s disease (n = 34)**	24	14	10	17

The healthy control individuals consisted of 17 men and 28 women (median age, 43 years; range, 28–75 years), mainly recruited during cancer screening programs. Regardless of previous antibiotic treatment, all case participants were tested for CDI according to routine guidelines. CDI was laboratory confirmed in 28 cancer patients and five IBD patients. All patients were enrolled before initiation of metronidazole or vancomycin treatment.

Clinical information was obtained from the institutional medical record management system with informed verbal consent. The stool samples were captured as discarded samples and stored at -80°C until use. All procedures were performed in accordance with the ethical standards of the institutional and/or national research committees, and with the 1964 Helsinki declaration and its later amendments or comparable ethical standards. The study was approved by the Ethics Committee of the Maria Sklodowska-Curie National Research Institute of Oncology (40/2018/1/2019).

### Methods

#### Metagenomics and metabolomics procedures

##### Metagenomic sequencing

Fecal DNA was extracted from 200 mg of feces using the QIAamp Fast DNA Stool Mini Kit protocol (Qiagen, Hilden, Germany) according to the manufacturer’s instructions (Kulecka et al., 2023). DNA was quantified using fluorimetry with the Qubit dsDNA High Sensitivity Assay (Thermo Fisher Scientific, Carlsbad, CA, USA). Shotgun metagenomic sequencing was performed using 10 ng of extracted DNA on the NovaSeq 6000 system (Illumina, San Diego, CA, USA) using 100 bps paired end reads following standard methods as provided by the manufacturer.

##### Short-chain fatty acid and amino acid profiling

Prior to analysis, 100 mg frozen stool sample was mechanically homogenized using 10% isobutanol and ceramic beads. Formate, acetate, propionate, butyrate, isobutyrate, and valerate, in addition to the amino acids alanine, L-arginine, L-cystine, L-glutamic acid, L-leucine, L-lysine, L-serine, L-threonine, L-tyrosine, L-valine, and L-histidine, were obtained from Sigma-Aldrich (St. Louis, MO, USA) for generation of calibration curves. Samples and calibration standards were derivatized with chloroformate isobutyl. Metabolites were extracted and derivatized as previously described (Kulecka et al., 2023).

Gas chromatographic analysis was performed using an Agilent 7000D Triple Quadrupole mass spectrometer coupled with a 7890 GC System with G4513A autosampler (Agilent Technologies, Santa Clara, CA, USA). MS data were analyzed using MassHunter software (Agilent Technologies, Santa Clara, CA, USA).

### Statistical analysis

Shannon diversity and Chao1 species richness indices (with confidence intervals) were calculated using the diversity function in the vegan package (version 2.5-7) (Dixon, 2003). Values were compared using the Kruskal-Wallis or Mann-Whitney U test (for two groups only). Bacterial taxa were assigned with Metaphlan3 (Beghini et al., 2023), version 3.0.13, using default parameters. Enterotypes were assigned according to the methods described by Arumugam et al. (Arumugam et al., 2011), using R code available at https://enterotype.embl.de/enterotypes.html. Fisher’s exact test was used to verify relationships between experimental groups and enterotypes. *Post hoc* analysis was performed according to methods described by Shan and Gerstenberger (Shan and Gerstenberger, 2017). Differences in taxon abundance between groups were assessed using the LINDA (LInear model for Differential Abundance) (Zhou et al., 2022) method for compositional data, and p-values were corrected using the Benjamini–Hochberg (Benjamini and Hochberg, 1995) method to minimize the false discovery rate (FDR). Differences in metabolite concentrations between study groups and enterotypes were assessed using the Kruskal-Wallis test (with an FDR-corrected Mann-Whitney U *post hoc* test).

*E. coli* virulence factors were identified with srst2 (Inouye et al., 2014), probing sequences from the virulence factors database (http://www.mgc.ac.cn/VFs/main.htm). Over-representation of GO biological processes was evaluated with ClueGo (Bindea et al., 2009).

Functional assignments were performed using human version 3.0 (part of BioBakery Workflows (Beghini et al., 2023)), using MetaCyc pathways (Caspi et al., 2020) as a reference database. Quality filtering and decontamination were performed with KneadData. The LINDA method was used to assess compositional data, with p-values corrected by the Benjamini–Hochberg procedure to minimize the FDR.

## Results

**Figure 1:**
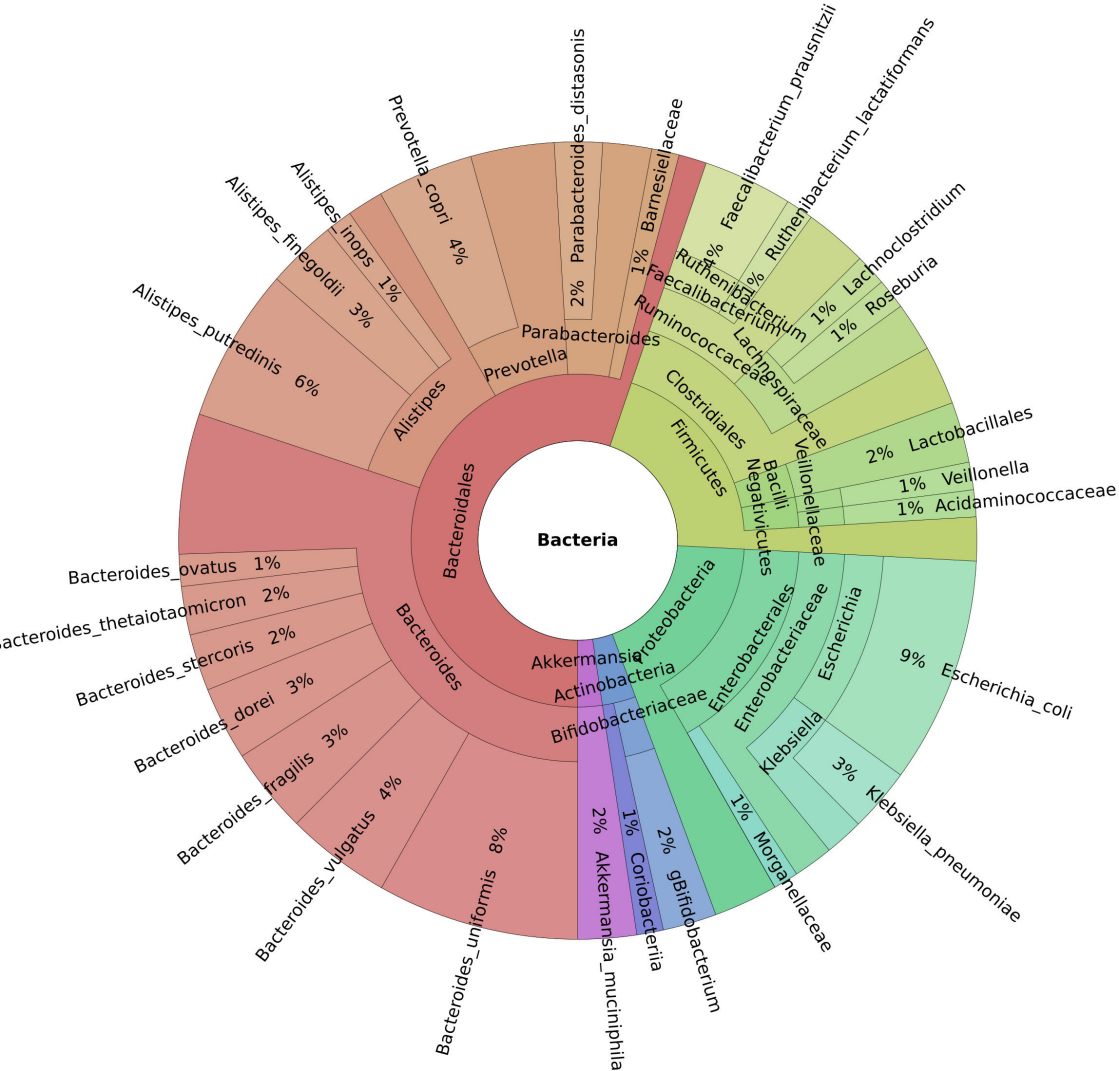
Krona chart of the genera with a mean abundance greater than 1% of the total.

This study investigated 138 cancer patients with healthcare-associated diarrhea and 43 patients with moderate or severe IBD. Of these, 27 cancer patients and five patients with IBD were also infected with *C. difficile*. The reference group consisted of 45 healthy controls. On average, 45 million reads were generated per sample (median 43 million). Five (*Bacteroidetes*, *Firmicutes*, *Proteobacteria*, *Actinobacteria*, and *Verrucomicrobia*) out of the 12 identified phyla had an abundance of more than 1% of the microbiome. Of the 194 genera discovered, 13 had a mean abundance greater than 1%, and *Bacteroides* were the most prevalent (**Figure 1**). Our dataset identified a total of 627 bacterial species.

### Bacterial community structure and enterotype-related changes

**Figure 2:**
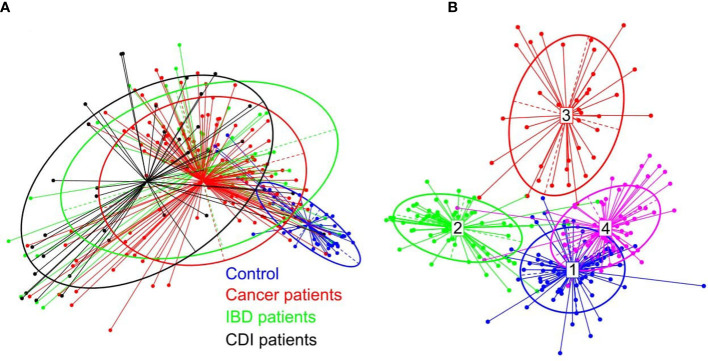
**(A)** β-diversity of bacterial structure evaluating similarities between the fecal microbiome population structure of the healthy control and patient groups, as determined by PCoA analysis based on a Bray-Curtis distance measure. **(B)** Between-class analysis visualization including four identified enterotypes.

β-diversity, evaluating similarities between the fecal microbiome population structure of the healthy control and patient groups, was determined using principal co-ordinate analysis (PCoA) of Bray-Curtis distances. Despite extensive variability between individual patients, the microbial compositions of cancer and IBD patients formed a single cluster, while healthy control and CDI samples formed two distinct clusters (**Figure 2A**).

In the healthy control group, we identified the three well-known enterotypes, of which enterotype 1 was driven by *Bacteroides*; enterotype 3 by *Prevotella*; and enterotype 4 by *Alistipes*, *Bacteroides*, and *Bifidobacterium* (**Figure 2B**). In the patient groups, we identified an additional enterotype driven by *Escherichia*. The enterotype frequencies differed between groups (**Table 3**). In healthy controls, *Escherichia*-driven enterotype 2 was not present, while the *Alistipes*-driven enterotype 4 was the most prevalent compared with all other participant groups (62% of samples). *Escherichia*-driven enterotype 2 reached 50% frequency in CDI patients and 30% in both cancer and IBD patients. Enterotype 1 was the most common in IBD and reached 40% frequency.

**Table 3:** Enterotype abundance in healthy controls and cancer, IBD, and CDI patients.

Groups	Enterotype (%)			
	1 (B)	2 (E)	3 (P)	4 (A)
**Healthy controls (n = 45)**	24	0	13	62
**Cancer patients (n = 43)**	29	29	20	22
**IBD patients (n = 119)**	40	30	9	21
**CDI patients (n = 38)**	24	50	8	18

(B) - Bacteroides-driven enterotype 1; (E) - Escherichia-driven enterotype 2; (P) - Prevotella-driven enterotype 3; Alistipes, Bacteroides, and Bifidobacterium-driven enterotype 4.

**Figure 3:**
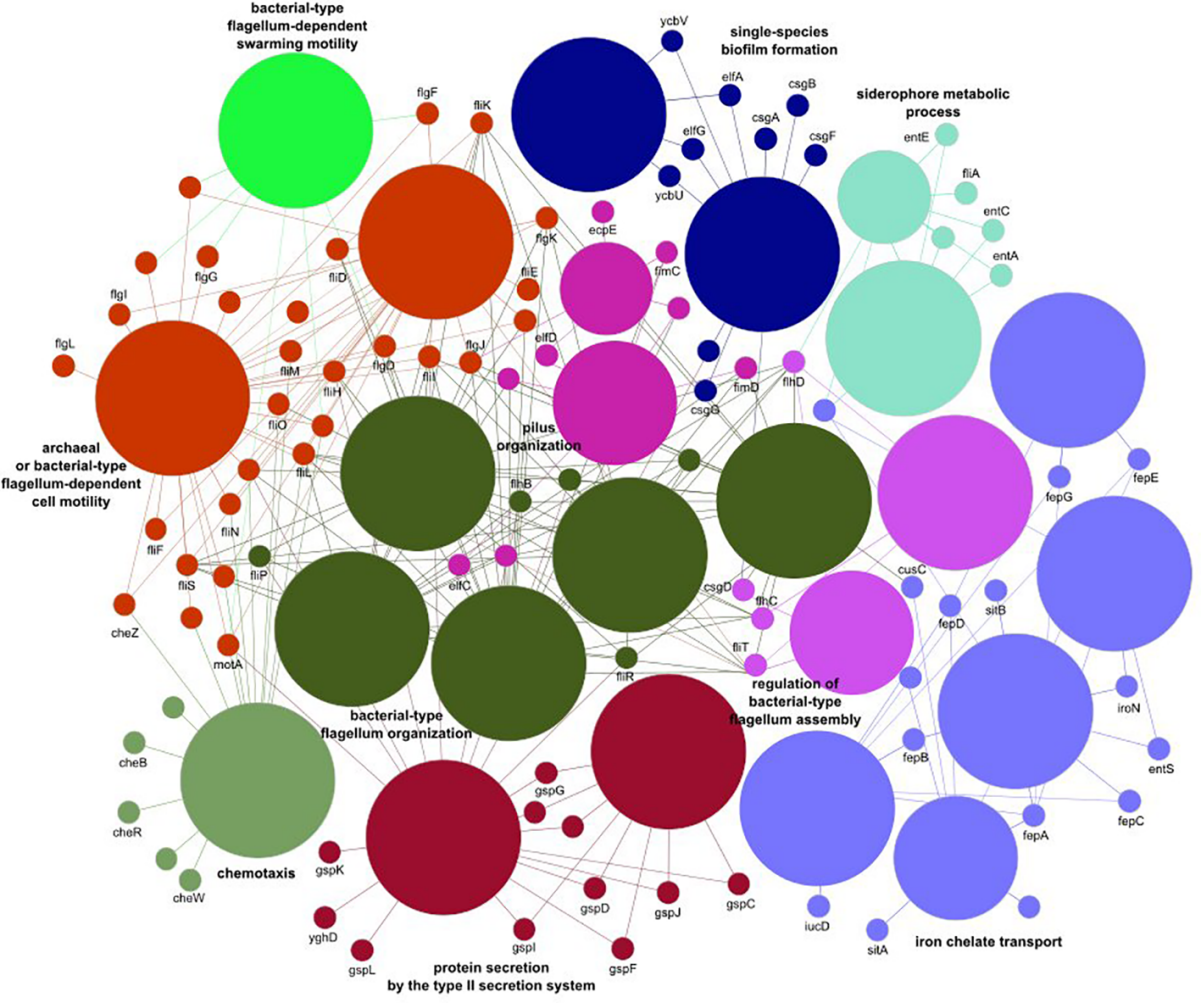
The *E. coli* virulence elements found to differ between healthy controls and cancer and *Clostridioides difficile* infected patients.

Noticeable levels of *E. coli* were detected in 55%, 67%, 88%, and 87% of fecal samples from healthy individuals, IBD patients, cancer patients, and CDI patients, respectively, with median gut microbiome abundances of 0.03, 0.59, 1.96, and 3.38, respectively (**Table 4**). Of the 496 *E. coli* analyzed virulence determinants, 268 were present in more than 10 samples. Although none of these virulence factors were differentially distributed in IBD patients compared with controls, 220 and 186 virulence factors were over-represented in cancer patients and CDI patients, respectively. Of these, 181 virulence factors were commonly distributed in the two pairwise comparisons. Of the genes defined as virulence factors from enteric *E. coli* pathotypes (Pakbin et al., 2021), only three (*eaeH*, *iutA*, and *csgA*), two (*fliR* and *fyuA*), and two (*fimA* and *ehaB*) genes were differentially distributed in CDI patients, cancer patients, and both groups of patients, respectively. The virulence factors over-represented jointly in both groups (**Supplementary Tables S1**-**S3**) were assigned to 25 biological processes belonging to 10 functional groups (**Supplementary Table S4**), including iron transport, biofilm formation, type II secretion, and cell motility (**Figure 3**).

**Table 4:** *Escherichia coli* abundances detected in healthy controls and cancer, IBD, and CDI patients.

Groups	*Escherichia coli*	
	Number of samples (percentage)	Median abundance (min–max)
**Healthy controls (n = 45)**	25 (55.55%)	0.03 (0–8.62)
**IBD patients (n = 43)**	29 (67.44%)	0.59 (0–97.66)
**Cancer patients (n = 119)**	105 (88.23%)	1.96 (0–86.07)
**CDI patients (n = 38)**	33 (86.84%)	3.38 (0–90.64)

### Bacterial α-diversity

**Figure 4:**
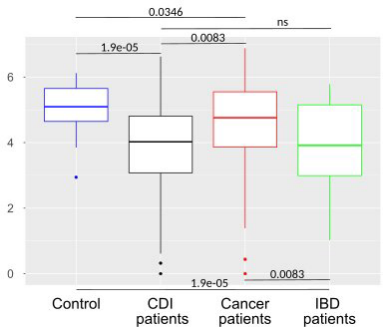
Bacterial α-diversity in samples from healthy controls, *Clostridioides difficile* infected patients, cancer patients, and patients with Inflammatory bowel disease measured by the Shannon index, a marker of richness and evenness.

**Figure 5:**
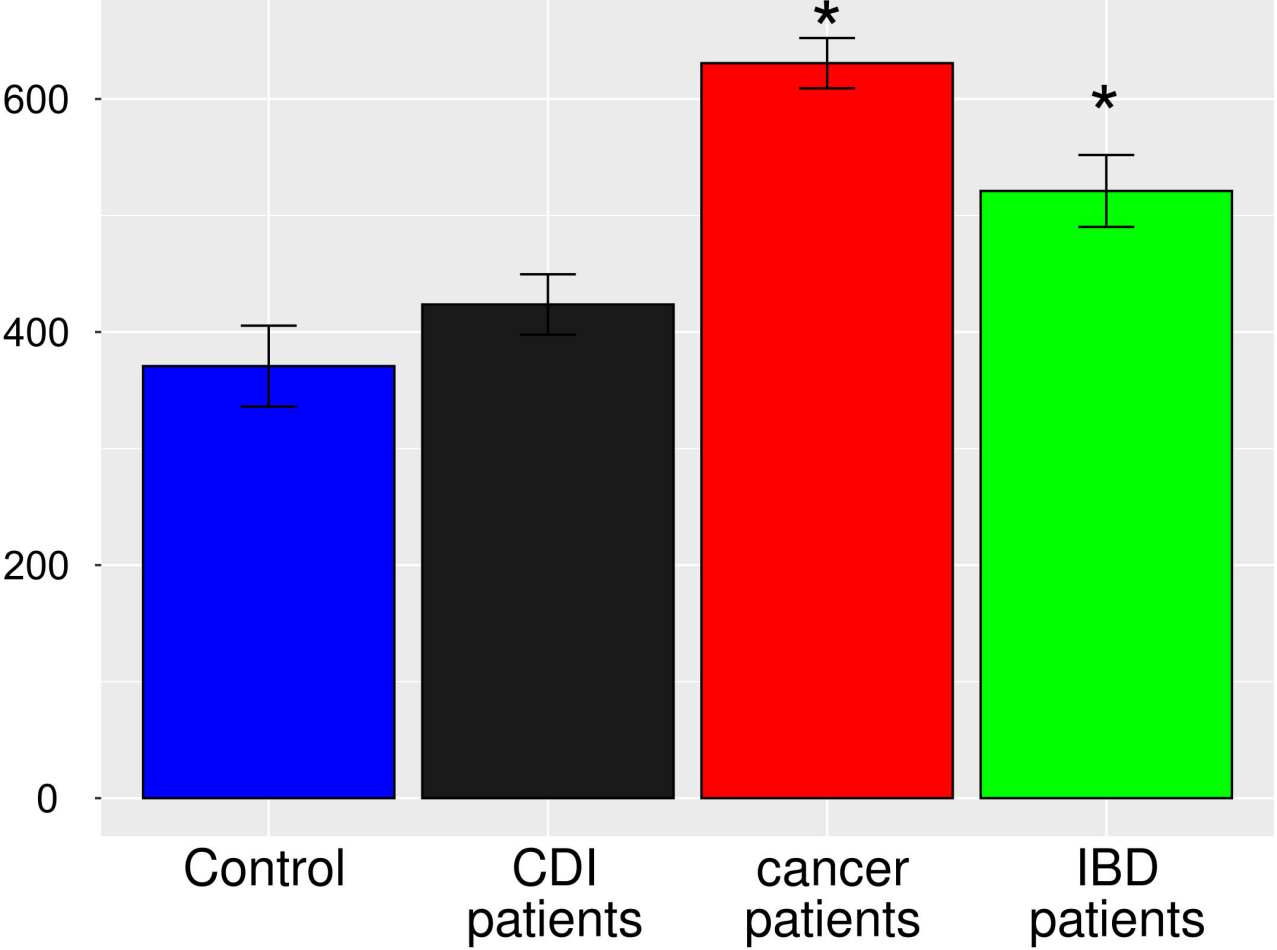
Bacterial α-diversity in samples from healthy controls, *Clostridioides difficile* infected patients, cancer patients, and patients with Inflammatory bowel disease, measured by the Chao1 index, a marker of bacterial richness. The Figure presents values with 95% confidence intervals; groups whose confidence intervals do not overlap with the control groups are marked with asterisk.

After multiple hypothesis testing corrections, α-diversity measured by the Shannon index, a marker of bacterial richness and evenness, was significantly lower in all groups than in healthy controls. Interestingly, the low the Shannon index was similar for IBD and CDI patients but was higher in cancer patients than in these two groups (**Figure 4**). The highest level of bacterial species richness, as measured by the Chao index, was observed in cancer patients, and statistically significant community dissimilarities were found between controls and cancer patients as well as CDI patients who were predominantly from the cancer group (**Figure 5**).

### Bacterial abundances

**Figure 6:**
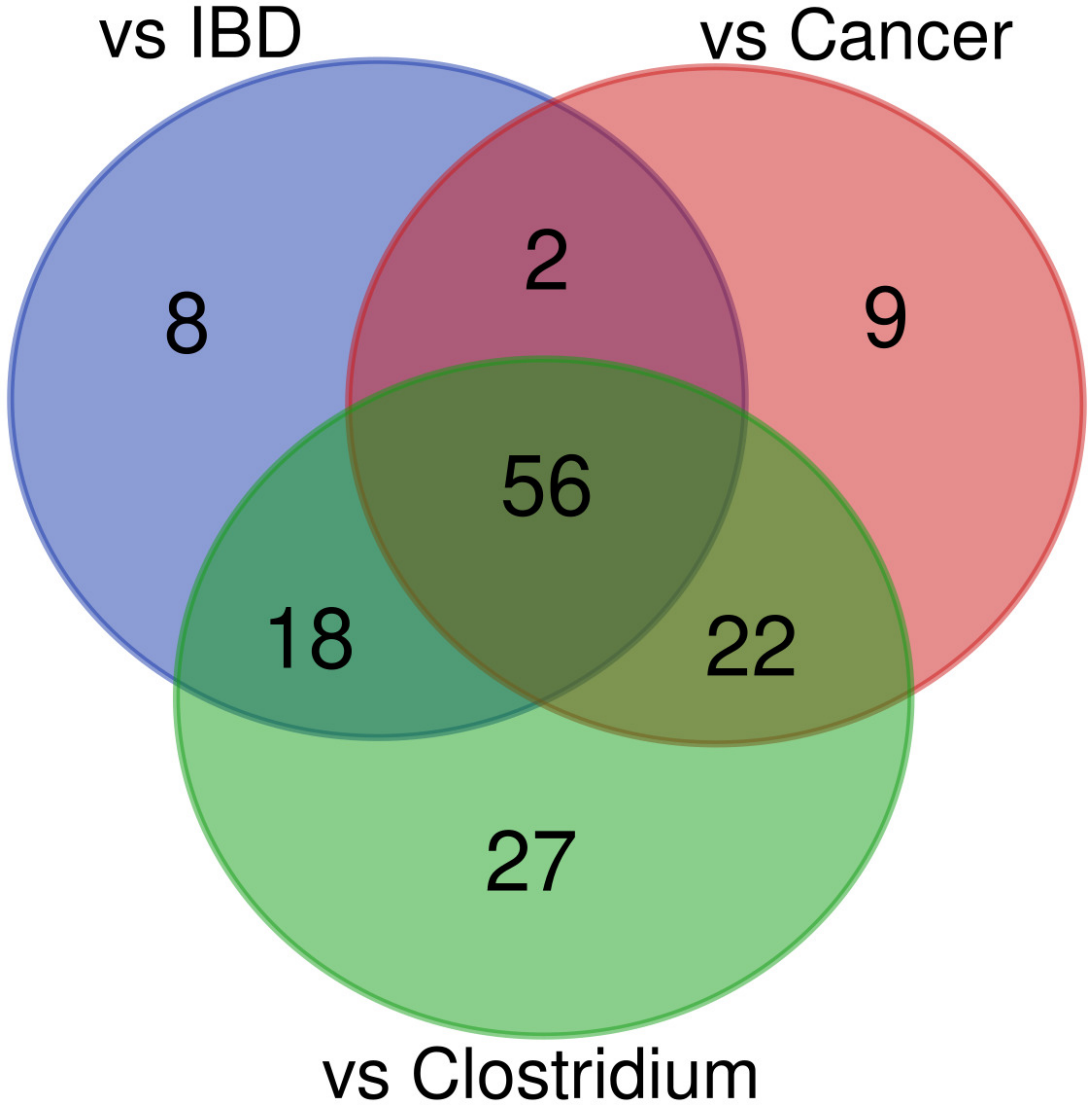
Venn diagram of bacteria found to differ between healthy controls and the patient groups.

The abundance of 89, 84, and 123 bacterial species was statistically different between healthy controls and uninfected patients with cancer, uninfected IBD patients, and *C. difficile* infected patients, respectively (adjusted p-value <0.05; **Supplementary Table S4** and **Figure 6**). To identify specific effects of CDI on diarrhea-associated intestinal dysbiosis, we searched for taxa that exhibit similarities between each of the group of patients compared with healthy controls and those that differ only between CDI patients and healthy controls. Together, 56 common species that differentiated each group of patients from healthy controls were selected, while the other 27 species specifically differentiated CDI patients from controls.

Of the 56 species whose abundance differentiated diarrheal patients from healthy controls, 52 and 4 species were under-represented and over-represented in diarrheal patients, respectively. Bacterial species with a lower abundance in diarrheal samples belonged to *Firmicutes* (n = 24), *Bacteroidetes* (n = 18), *Actinobacteria* (n = 6), *Proteobacteria* (n = 3), and *Lentisphaerota phylla* (n = 1). Most species were normal anaerobic commensals of the healthy human gut, and only five species (*Agathobaculum butyriciproducens*, *Lawsonibacter asaccharolyticus*, *Ruthenibacterium lactatiformans*, *Bilophila wadsworthia*, and *Desulfovibrionaceae bacterium*) were obligate anaerobes. Instead, four species (*Bacteroides fragilis*, *Escherichia coli*, *Klebsiella pneumoniae*, and *Ruminococcus gnavus*) with a higher abundance in diarrheal samples than in healthy controls may be considered opportunistic pathogens, and two of them are obligate anaerobes.

Of the 27 species that specifically differentiated CDI samples, 8 and 19 species were under-represented and over-represented, respectively. As before, they were mainly part of the normal human gut flora. All under-represented species were anaerobes, while one over-represented bacteria was facultatively anaerobic, and the other was microaerophilic.

Using the MetaCyc Metabolic Pathway Database, which allows the reconstruction of metabolic networks from sequenced genomes, we identified 495 MetaCyc pathways, of which 425 met criteria for statistical analysis. Of these, 185 pathways differentiated all tested groups from healthy controls and consisted of categories including cofactor biosynthesis, energy metabolism, degradation of aromatic compounds, and lipid and amino acid biosynthesis (**Supplementary Table S6**). We also identified pathways implicated in bacterial pathogenesis, including bacteriocin synthesis and antibiotic resistance. Nineteen, 21, and 11 pathways were unique to patients with *C. difficile*, cancer, and IBD, respectively. In cancer patients, these pathways were associated with lipid metabolism, while in CDI patients, they were associated with energy metabolism.

### Fecal SCFA and amino acid profiling

GC/MS-based analysis of fecal samples identified eight SCFAs (acetic acid, butanoic acid, formic acid, hexanoic acid, isobutyric acid, isocaproic acid, pentanoic acid, propanoic acid) and nine amino acids (alanine (Ala), glycine (Gly), glutamic acid (Glu), isoleucine (Ile), leucine (Leu), methionine (Met), phenylalanine (Phe), proline (Pro), and valine (Val)).

**Figure 7:**
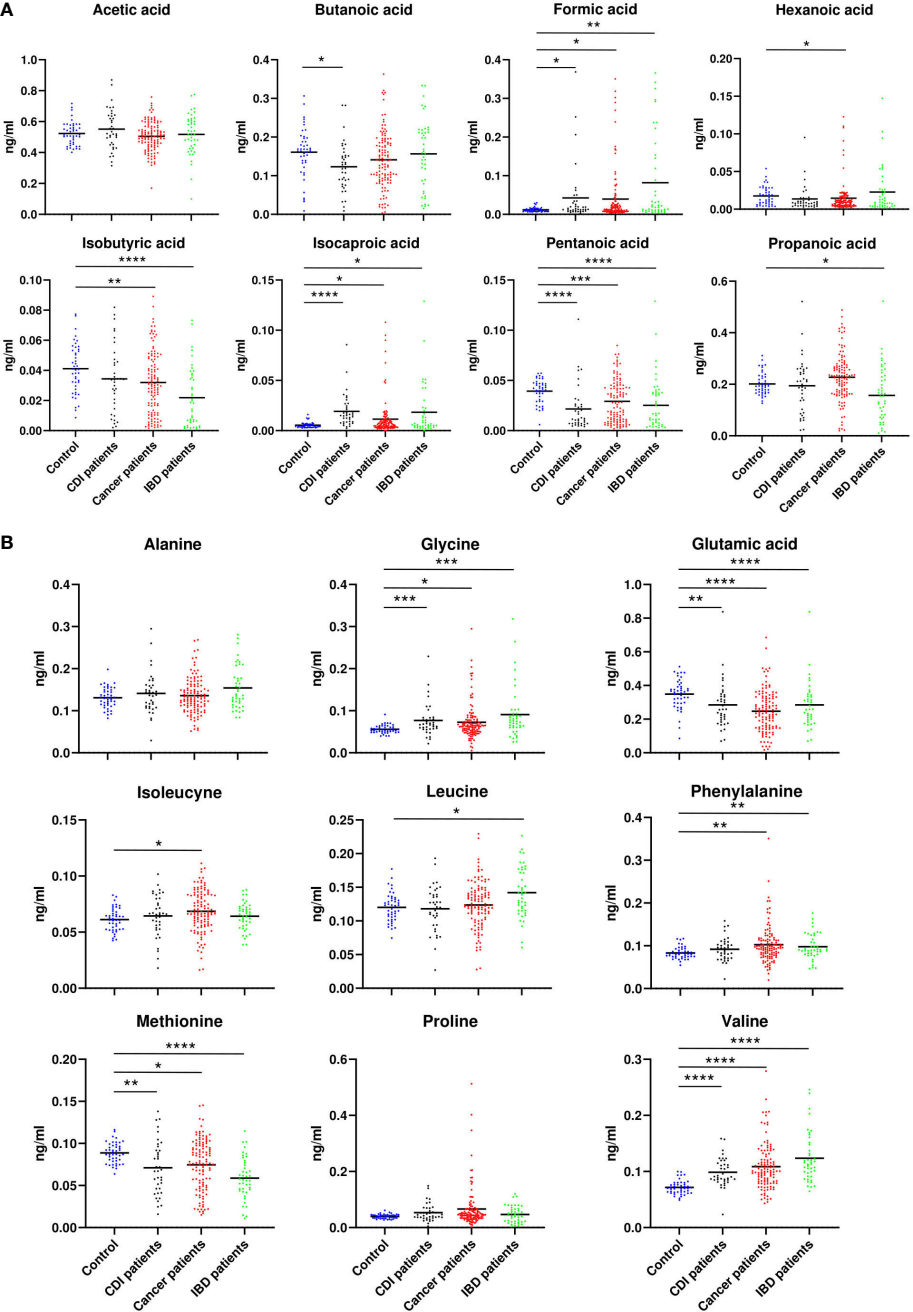
Relative abundance of SCFA **(A)** and amino acids **(B)**, which differed between patients with cancer, inflammatory bowel disease, or *Clostridioides difficile* infected and healthy control individuals. Statistical significance was determined using a Mann-Whitney U test. *; p ≤ 0.05, **; p ≤ 0.01, ***; p ≤ 0.001, ****; p ≤ 0.0001. Vertical bars indicate the mean.

No association was found between acetate and each of the diarrheal patient groups., Hexanate was over-represented in cancer patients (p=0.02). Isobutyrate was under-represented in samples from cancer (p=0.009) and IBD patients (p=0.00001), propanate was under-represented in IBD patients (p=0.001), and butyrate was under-represented in CDI patients (p=0.001, **Figure 7A**). The relative abundance of Ala and Pro did not differ between the nondiarrheal controls and any of the patient groups. Instead, Gly (p=0.0002, p=0.01 and p=0.0004) and Val (p<0.00001) were over-represented, and Met (p<0.00001,p=0.02,p=0.004) and Glu (p<0.00001,p=0.02, p<0.00001,p=0.002) were under-represented, in each of the three groups of diarrheal patients (p-values given for IBD, cancer and CDI patients respectively). Additionally, an over-representation of Phe was found in both cancer patients and IBD patients (p=0.005 and p=0.009), and Ile (p=0.03) and Leu (p=0.01) were over-represented in cancer and IBD patients, respectively (**Figure 7B**).

### Correlation analysis between bacteria and metabolites

To evaluate association between the 56 and 27 species abundances that differentiated all diarrheal patients from healthy controls and only CDI patients from controls, respectively, and the levels of SCFAs and amino acids, we used the Spearman coefficient. Of four species that differentiated diarrheal individuals from nondiarrheal individuals, *Ruminococcus gnavus*, *E. coli*, and *Klebsiella pneumoniae* correlated negatively with pentanoic acid concentrations (coefficients -0.26, -0.23 and -0.28 respectively); *E. coli* and *Klebsiella pneumoniae* correlated negatively with Glu (coefficients: -0.28 and -0.24) and positively with Val (coefficients: 0.38 and 0.3); and *Ruminococcus gnavus* and *E. coli* correlated positively with Phe (coefficients: 0.21 and 0.26). Most under-represented species in diarrheal cases correlated negatively with formic acid, isocaproic acid, Gly, and Val, and positively - with isobutyric acid, butanoic acid, and pentanoic acid.

Of eight species under-represented in CDI patients compared with healthy controls, *Slackia isoflavoniconvertens*, *Blautia obeum*, *Ruminococcus torques*, *Dorea longicatena*, and CAG 139 correlated negatively with isocaproic acid (coefficients: -0.23, -0.26, -0.22, -0.33 and -0.21 respectively). *Eubacterium ramulus*, *Blautia obeum*, and *Dorea longicatena* correlated positively with butanoic acid (coefficients: 0.22, 0.21 and 0.33 respectively). *Bacteroides dorei*, *Blautia obeum*, and *Ruminococcus torques* correlated with Met abundance. Of the 19 species over-represented in CDI patients, *Enterococcus faecalis*, *Lactobacillus rhamnosus*, and *C. difficile* positively correlated with isocaproic acid (coefficients: 0.2, 0.25 and 0.26 respectively), while other metabolites mostly correlated with individual bacteria.

## Discussion

A low diversity microbiota and increased proportions of facultative anaerobes are typically associated with diarrhea of different etiologies, including CDI, IBD, and systemic cancer therapy. This metagenomic study aimed to define the population structures of the fecal microbiota of diarrheal case-mix cancer patients, patients with IBD, and normal healthy individuals. Patient groups were further classified according to the presence of CDI.

PCoA defined three distinct clusters that corresponded to the four different phenotypic groups (**Figure 2**). Of these, CDI-negative cancer and IBD patients clustered separately from both healthy individuals and combined CDI positive cases (**Figure 2A**). α-diversity was significantly lower in all diarrheal groups compared with healthy controls. The lowest α-diversity was found in IBD and CDI groups (**Figure 4**), and the highest bacterial species richness was observed in cancer patients (**Figure 5**). Despite high variability between individual groups, the gut microbiota of CDI-negative cancer and IBD patients was highly dysbiotic, and this was further exacerbated by CDI. We identified three ‘healthy’ enterotypes among nondiarrheal individuals, of which the *Alistipes*-driven enterotype was detected in 62% of the control samples. Instead, in one third of uninfected diarrheal patients and half of the CDI patients, we identified an additional *Escherichia-*driven enterotype. CDI-negative cancer and IBD patients showed the greatest enterotype concordance, with similarities to the composition of CDI-related enterotypes. Together, categorizing enterotypes improved the identification of specific diarrheal states. Our data are in agreement with previous studies that reported these enterotypes in diarrheal children (Mobeen et al., 2018) and colorectal cancer patients (Schubert et al., 2014; Milani et al., 2016; Vandeputte et al., 2016; Jiang et al., 2017; Zaneveld et al., 2017; Aarnoutse et al., 2019; Yang et al., 2019; Ervin et al., 2020; Ke et al., 2021; Wang et al., 2021; Chung The and Le, 2022).

Reduced diversity is not always indicative of reduced total bacterial numbers. A balanced gut microbiota promotes colonization resistance to diarrheal pathogens, but dysbiosis may favor the proliferation of fast-growing facultative anaerobes (Maier et al., 2021). We identified 89, 84, and 123 species whose relative abundances differentiated healthy individuals from uninfected cancer patients, uninfected IBD patients, and CDI patients, respectively. Of these, 52 and 4 species (*Escherichia coli*, *Bacteroides fragilis*, *Klebsiella pneumoniae*, and *Ruminococcus gnavus*) were reduced and enriched in each of the three pairwise comparisons between patient groups and the control group, respectively. Although under-representation of taxa can be explained by lower overall bacterial diversity, over-representation of opportunistic pathogens may result from reduced niche competition during dysbiosis, leading to the proliferation of pathobionts (Milani et al., 2016; Yu, 2018; Chandra et al., 2021).

*E. coli* is the predominant aerobic constituent of the gut microbiota, which is implicated in opportunistic intestinal and extraintestinal infections (Denamur et al., 2021). Seven pathotypes, including enteropathogenic *E. coli* (EPEC), enterohemorrhagic *E. coli* (EHEC/STEC), enteroaggregative *E. coli* (EAEC), enterotoxigenic *E. coli* (ETEC), and enteroinvasive *E. coli* (EIEC), have been described, and their pathogenesis is driven by specific gene clusters. Individual virulence factors can also be shared by different pathotypes (Jesser and Levy, 2020; Pakbin et al., 2021).

Previously, increased abundances of *Escherichia* were found in patients following chemotherapy. This abundance gradually increased during chemotherapy and correlated with onset of IBD (Aarnoutse et al., 2019). Using shotgun metagenomic sequencing, our study revealed an increased abundance of *E. coli* in all three groups of diarrheal patients compared with normal individuals. However, while 220 and 186 genomic virulence factors of *E. coli* were found to be over-represented in cancer patients and CDI patients, respectively, no virulence factor was differentially distributed between patients with IBD and controls. Of these, 181 virulence elements were commonly distributed in both pairwise comparisons between cancer or CDI patients and controls. Of the differentially distributed genes in cancer and CDI patients, only a few were previously reported to encode virulence factors of diarrheagenic *E. coli* (DEC) pathotypes (Pakbin et al., 2021). Therefore, either the *E. coli* strains identified in this study do not exhibit genetic signatures of DEC or such pathotypes are difficult to detect. Indeed, DEC pathotypes are poorly represented in sequencing databases (Jesser and Levy, 2020).

*Bacteroides fragilis* is an obligate anaerobe with pathogenesis driven by the enterotoxin fragilysin, which has been studied in the context of diarrheal patients, IBD patients, and colorectal cancer patients (Toprak et al., 2006; Wick and Sears, 2010). *Klebsiella pneumoniae* is a common opportunistic pathogen from the *Enterobacteriaceae* family, which colonizes the human respiratory and intestinal tract. *K. pneumoniae* can cause severe nosocomial and community-acquired infections including pneumonia, urinary tract infections, and occasionally diarrhea (Lu et al., 2017; Zhang et al., 2018). *Ruminococcus gnavus* has been identified as a key risk factor for diarrhea-predominant irritable bowel syndrome IBS-D (Han et al., 2022; Zhai et al., 2022). Diarrhea-related low diversity dysbiosis is associated with distinct taxa including *Bacteroides*, *Lachnospiraceae*, *Ruminococcaceae Porphyromonadaceae*, *Ruminococcus*, *Roseburia*, *Blautia*, *F. prausnitzii*, and *B. fragilis* (Schubert et al., 2014), which are usually of transient and/or low abundance (Chung The and Le, 2022). Conversely, an increase in the abundance of *Clostridiales*, *Citrobacter*, *Klebsiella*, *Enterococcus*, *Megasphaera*, *Ruminococcus* sp., and *Parabacteroides* is observed following systemic cancer therapy (van Vliet et al., 2009; Zwielehner et al., 2011; Montassier et al., 2014; Montassier et al., 2015; Gopalakrishnan et al., 2018; Routy et al., 2018; Fei et al., 2019).

Our findings confirmed that under dysbiosis, usually harmless pathobionts, such as *C. difficile*, *Helicobacter pylori*, segmented filamentous bacteria (SFB), invasive *Escherichia coli*, *Proteus mirabilis*, *Klebsiella pneumoniae*, *Prevotellaceae*, TM7, and vancomycin-resistant *Enterococcus* spp., may proliferate and drive pathogenesis (Chandra et al., 2021). Indeed, *C. difficile* colonizes the gastrointestinal tracts of 2–5% of healthy adults prior to dysbiosis (Binder, 2010; Rood et al., 2018).

Low diversity dysbiosis is driven by many factors, including diarrhea of different etiologies. For example, in cancer patients, diarrhea can have infectious and noninfectious etiologies, including cytotoxic mucosal injury and indirect antibiotic-related changes of the gut bacterial community (Ervin et al., 2020; Shi et al., 2020; Wang et al., 2021). The incidence of chemotherapy-induced diarrhea has been reported as high as 50–80% in patients undergoing systemic therapy (Ervin et al., 2020) due to increased intestinal permeability and modulation of the gut microbiota. Some chemotherapeutics induce neutropenic enterocolitis, ischemic colitis, and colitis associated with *C. difficile*, while immune checkpoint inhibitors may promote immune-mediated colitis (Ervin et al., 2020). As reviewed recently (Wang et al., 2021), pelvic radiotherapy can lead to low diversity intestinal dysbiosis, which is exacerbated in patients with radiation therapy-induced diarrhea. Radiation-induced enteritis affects 80% of patients treated for anorectal, gynecological, or prostate cancers (Tonneau et al., 2021).

*Roseburia* and *Bifidobacterium*, which are considered healthy, anti-inflammatory constituents of the gut microbiota, decrease in frequency after treatment (Montassier et al., 2014). The majority of diarrheal cancer patients enrolled in our study were undergoing systemic cancer therapy, which could be a major cause of diarrhea-related intestinal dysbiosis. Instead, low diversity intestinal dysbiosis in IBD patients is a key factor in the complex interaction between IBD genetic risk and dysregulation of the innate and adaptive immune systems (Boland et al., 2021). Indeed, intestinal bacterial taxa have been identified as major contributors to the clinical status of CD patients (Ambrozkiewicz et al., 2020). IBD phenotypes generally relate to changes in the bacterial population structure and combinations of specific taxa, rather than alterations of individual species abundance (Boland et al., 2021).

CDI is a common problem in cancer patients, primarily driven by the high rate of oral antibiotic use and hospitalization (Ervin et al., 2020). This is likely due to alteration in intestinal microbiota function and structure, which promotes colonization by *C. difficile* and other enteric pathogens (Ross et al., 2016). In contrast to the increased dissimilarity of the bacterial communities observed from patients with CDI without recurrence, both CDI recurrence and reinfection are associated with a decrease in α-diversity at the initial diagnosis of CDI (Seekatz et al., 2016). About 10% of patients enrolled in our study with diarrheal cancer and IBD were also affected by CDI.

Many taxa differed between CDI patients and nondiarrheal individuals. Of these, 27 species (8 under-represented and 19 over-represented) accurately differentiated CDI patients from healthy controls. These species represented normal human gut flora, with the exception of *C. difficile*. All under-represented species were anaerobes, while one over-represented bacteria was facultatively anaerobic, and the other was microaerophilic. These data provide additional evidence that CDI exacerbates diarrhea-related dysbiosis, but it remains unclear whether CDI-associated dysbiosis is a consequence of infection or whether CDI is the causative agent of dysbiosis.

CDI-related infectious diarrhea occurs in 20% of patients receiving broad-spectrum antibiotics (Binder, 2010). Most cases of antibiotic-associated diarrhea occur in response to a decrease in the levels of butyrate-producing anaerobic bacteria. Furthermore, chemotherapy reduces the abundance of SCFA-producing bacteria (Aarnoutse et al., 2019). Decreased production of butyric acid and other SCFAs subsequently promotes accumulation of luminal carbohydrates and colonic bile acids, increasing osmotic load and water absorption within the intestinal lumen, eventually causing diarrhea (Binder, 2010; Machiels et al., 2014; Theriot and Young, 2014; Mekonnen et al., 2020). Considering these observations, our patient cohort likely had variable mechanisms underpinning their diarrhea.

**Figure 8:**
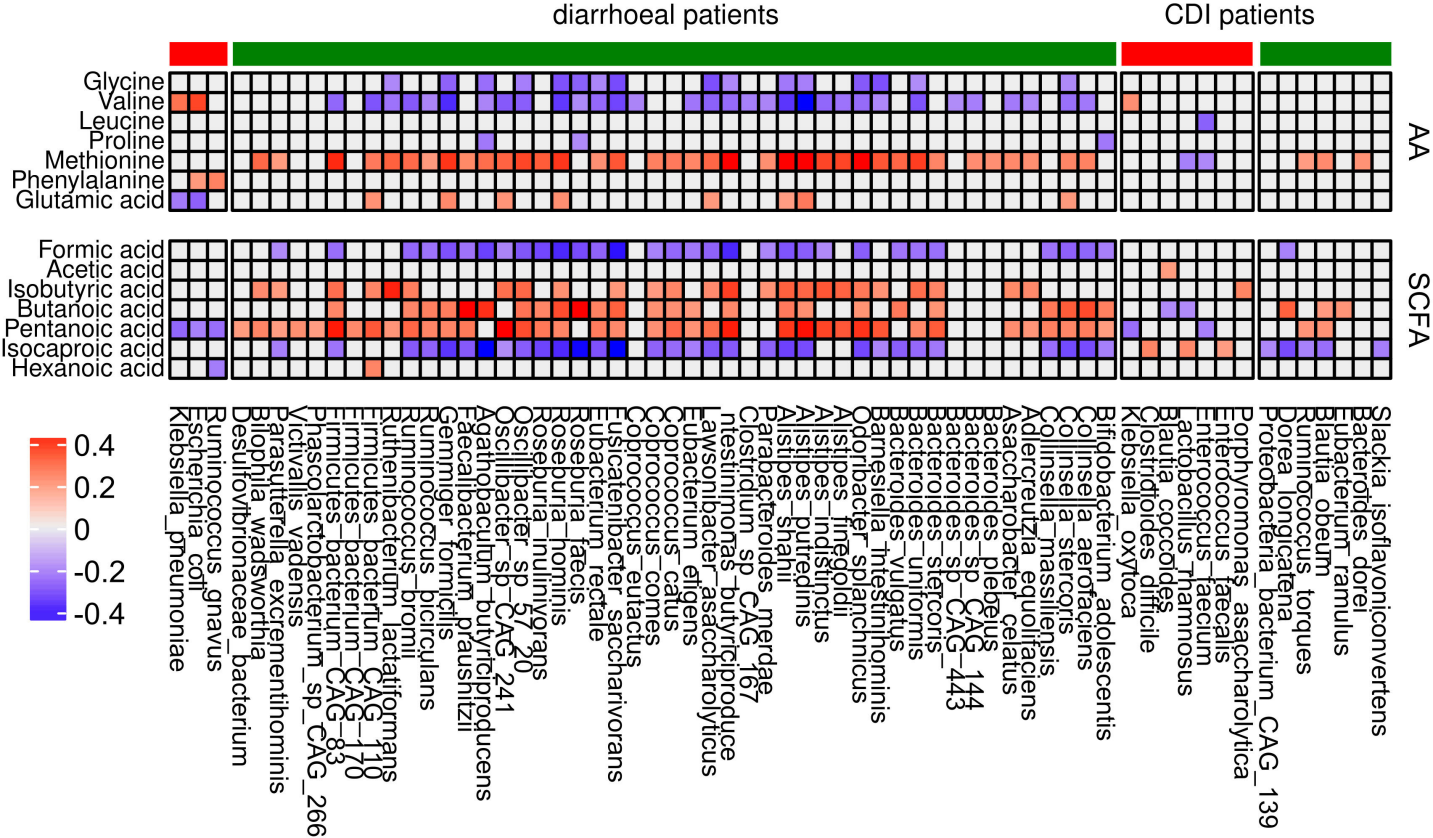
Correlation between differential bacterial species abundance and concentrations of amino acids and SCFAs. Only coefficients with absolute values higher than 0.2 are shown. Red and green denote bacteria that are over- and under-represented, respectively, in each group of patients in comparison with the control group.

SCFAs are a large group of fecal microbial metabolites present in the intestinal lumen at millimolar concentrations. Acetate, propionate, and butyrate are present in molar ratios of approximately 60:20:20 (Deleu et al., 2021). In this study, we found that the relative abundance of seven out of nine assayed fecal SCFAs differentiated at least two groups of diarrheal patients from healthy controls. Formic acid and caproic acid were more abundant, and pentanoic acid was less abundant, in each of the three diarrhea groups. Five amino acids differentiated at least two patient groups from healthy controls. Of these, glycine and valine were more abundant, and methionine and glutamic acid were less abundant, in each patient group. Abundance of the 56 species that differentiated patient groups from controls correlated with concentrations of formic, isobutyric, butyric, pentanoic, and isocaproic acid, as well as with those of glycine, valine, and methionine. Abundance of many of the 27 species that differed between patient groups and healthy controls correlated with concentrations of butyric, pentanoic, and isocaproic acid and, in some cases, methionine (**Figure 8**).

SCFAs modulate the intestinal barrier by stimulating mucus production by IECs, altering the arrangement of tight junction proteins, and interacting with the systemic immune system after translocation from the gut to the bloodstream (Deleu et al., 2021). Our previous study revealed that butyrate and valerate levels differed significantly between CD patients and controls, and that propionate, butyrate, isobutyrate, and valerate levels differed between CD patients with active and inactive disease (Deleu et al., 2021). A decrease in acetate and propionate concentrations, but not in that of butyrate, was observed in UC patients (Machiels et al., 2014), while another study showed a decrease in propionate and butyrate (Huda-Faujan et al., 2010). In this study, butyrate differentiated only CDI patients and healthy individuals. Further work is required to establish the relevance of SCFAs as modulators of diarrhea.

Both bile salts and amino acids play an important role in the *C. difficile* life cycle. Glycine in combination with certain bile acids promotes *C. difficile* germination, which can be further enhanced by histidine, arginine, aspartic acid, and valine. Tryptophan was also demonstrated to play a role in colonization resistance (Ross et al., 2016). Amino acids in the intestine regulate the expression of tight junction proteins and are linked to IEC apoptosis and proliferation (Zhang et al., 2019). Tryptophan, phenylalanine, and tyrosine may attenuate gastrointestinal inflammation, and concentrations of tryptophan and histidine are significantly lower in patients with UC compared with controls (Xin et al., 2015; Tan et al., 2017).

Overall, our study confirmed that low diversity dysbiosis in combination with changes to microbiome population structure are a consequence of systemic cancer therapy. However, similar changes were also found in IBD patients and in those with CDI. Therefore, different external and internal factors can drive low diversity dysbiosis with similar phenotypes. Here, we tested a case mix of diarrheal cancer patients with a wide range of gastrointestinal cancers and nongastrointestinal neoplasms, undergoing different treatment regimens. Heterogeneity of both cancer type and treatment regimen can be considered both a strength and a limitation of our study. The other limitations included the small sample size, particularly small number of IBD patients infected with *C. difficile*; the exploratory nature of the study which did not provide mechanistic insights into the relationship between the microbiome and intestinal injury, as diagnostic procedures for causes of therapy-related diarrhea were limited to only CDI; and limited knowledge about lifestyle and dietary habits that could influence microbiota composition. In fact, complexity of intestinal microbiome structure and function still remains a challenge for researchers, as multiple confounding factors preclude assignment of causal relationships between the microbiome and pathology.

The practical use of intestinal dysbiosis analyses in predicting the further cancer development at the present stage of our knowledge is rather unlikely. Despite this, we believe our data provide a mandate for further research on the causal relationship between low diversity dysbiosis and systemic cancer therapies, including chemotherapy, hormone therapy, targeted drug therapy and immunotherapy. Future studies will compare the structures of gut dysbiosis-associated microbiomes and gut metabolite abundances in cancer patients receiving systemic therapies, with and without treatment-related diarrhea and other cancer-related side effects.

## Data availability statement

The datasets presented in this study can be found in online repositories. The names of the repository/repositories and accession number(s) can be found below: https://www.ncbi.nlm.nih.gov/, PRJNA943135.

## Ethics statement

The studies involving human participants were reviewed and approved by Bioethics Committee at the Maria Sklodowska-Curie National Research Institute of Oncology (40/2018/1/2019). The patients/participants provided their written informed consent to participate in this study.

## Author contributions

MK: data curation, formal analysis, visualization, writing–original draft, writing–review and editing. NZ-L: investigation, visualization, and writing–original draft, writing–review and editing, project administration. AB, PC, KB, MG, AK, MP, MD: investigation. EW: resources. MM: visualization and writing–review and editing. JO: conceptualization, funding acquisition, project administration, writing–original draft, and writing–review and editing. All authors contributed to the article and approved the submitted version.
